# An SK3 Channel/nWASP/Abi-1 Complex Is Involved in Early Neurogenesis

**DOI:** 10.1371/journal.pone.0018148

**Published:** 2011-03-25

**Authors:** Stefan Liebau, Julie Steinestel, Leonhard Linta, Alexander Kleger, Alexander Storch, Michael Schoen, Konrad Steinestel, Christian Proepper, Juergen Bockmann, Michael J. Schmeisser, Tobias M. Boeckers

**Affiliations:** 1 Institute for Anatomy and Cell Biology, Ulm University, Ulm, Germany; 2 Department of Internal Medicine I, Ulm University, Ulm, Germany; 3 Institute of Molecular Medicine and Max-Planck-Research Group on Stem Cell Aging, Ulm, Germany; 4 Department of Neurology and Center for Regenerative Therapies Dresden (CRTD), Dresden University of Technology, Dresden, Germany; University of Nebraska Medical Center, United States of Amrica

## Abstract

**Background:**

The stabilization or regulated reorganization of the actin cytoskeleton is essential for cellular structure and function. Recently, we could show that the activation of the SK3-channel that represents the predominant SK-channel in neural stem cells, leads to a rapid local outgrowth of long filopodial processes. This observation indicates that the rearrangement of the actin based cytoskeleton via membrane bound SK3-channels might selectively be controlled in defined micro compartments of the cell.

**Principal Findings:**

We found two important proteins for cytoskeletal rearrangement, the Abelson interacting protein 1, Abi-1 and the neural Wiskott Aldrich Syndrome Protein, nWASP, to be in complex with SK3- channels in neural stem cells (NSCs). Moreover, this interaction is also found in spines and postsynaptic compartments of developing primary hippocampal neurons and regulates neurite outgrowth during early phases of differentiation. Overexpression of the proteins or pharmacological activation of SK3 channels induces obvious structural changes in NSCs and hippocampal neurons. In both neuronal cell systems SK3 channels and nWASP act synergistic by strongly inducing filopodial outgrowth while Abi-1 behaves antagonistic to its interaction partners.

**Conclusions:**

Our results give good evidence for a functional interplay of a trimeric complex that transforms incoming signals via SK3-channel activation into the local rearrangement of the cytoskeleton in early steps of neuronal differentiation involving nWASP and Abi-1 actin binding proteins.

## Introduction

Neurons of the central (CNS) as well as of the peripheral nervous system undergo dramatic structural changes especially throughout early stages of brain development [1], [2], [3]. Especially the formation and plasticity of spines and synapses is highly dynamic throughout the entire lifespan and are thought to explain learning and memory formation within the CNS [4], [5].

However, neurogenesis is taking place also in the adult brain. In several subcompartments of the CNS, neural stem cells (NSCs) give rise to new neurons upon specific stimuli [6]. As during embryonic development, these stem cells need to migrate, differentiate and integrate in order to be part of the functional nervous tissue. To execute structural changes, the controlled rearrangement of cytoskeletal components in small cellular subcompartments plays a pivotal role [7], [8], [9], [10]. It has been shown that the rearrangement machinery consists of several protein complexes that are responsible for distinct functions [11]. According to the local task, cytoskeletal proteins themselves interact with a variety of molecules including motor proteins or members of specific signaling pathways [12], [13].

The actin based cytoskeleton is most dynamic part of the cytoskeleton. Within microcompartments like filopodia and lamellipodia which are important for migration, integration into a cellular network and differentiation of newly generated neurons as well as within specialized neuronal structures like synaptic spines immediate, fast and controlled changes of actin filaments are needed [7], [10], [14], [15]. Actin is built of the g-actin molecules (globular) which can self-assemble depending on e.g. abundance of g-actin, pH or membrane potentials [16], [17]. Regulating proteins on the other hand can promote or prevent elongation, branching or disruption of actin filaments. Well known molecules in these complexes are proteins like Cdc42, Arp2/3, Cofilin, nWASP, Abi-1 or Fascin [18].

Membrane spanning proteins, e.g. ligand depending receptors and ion channels can guide extrinsic signals to these protein complexes [19], [20]. Expression, localization and specific activation of different ion-channels are known to be essential during development and maturation of undifferentiated stem and progenitor cells. During these processes cell morphology is characterized by the dynamic formation and reorganization of small cellular compartments of the outer cell structure like filopodia and lamellipodia. The structural basis are cytoskeletal proteins that are organized as dynamic macromolecular complexes [14] and their modulation depends on the activation of ion channels [21], [22], [23]. Especially Ca^2+^-activated voltage independent K^+^ channels (K_Ca_ channels) influence the reorganization of lamellipodia [21], [24] and dendritic spines [23]. Recently, we could show that SK3 channel activity induces the alteration of stem cell morphology [19]. However, it remained elusive how the external signal is transformed.

SK3 channels are densely localized in the filopodial and lamellipodial micro compartments [19]. As these channels do not transduce large ion potentials, they are a good candidate for signaling pathways that take place in a micro compartment. SK-channels (K_Ca_) form two subfamilies, small and intermediate conductance K^+^ channels (SK1-3, IK), consisting of four members, SK1 (K_Ca_2.1, KCNN1), SK2 (K_Ca_2.2, KCNN2) SK3 (K_Ca_2.3, KCNN3) and SK4 (IK, K_Ca_3.1, KCNN4). They are activated upon elevated intracellular Ca^2+^-concentrations. Ca^2+^ is the only known physiological activator of K_Ca_-channels and once activated, they can be kept in an open conformation by 1-ethyl-2-benzimidazolinone (EBIO) and its derivatives (e.g. DC-EBIO) [25].

EBIO, which enhances the activity of SK-channels by increasing their apparent Ca^2+^-sensitivity, has been proven to be valuable for investigating SK-channel physiology [26]. Calmodulin constitutively binds to SK-channels and functions as a Ca^2+^-sensor. In response to Ca^2+^-binding to calmodulin, channel opening occurs with time constants of activation of 5–15 ms [27]. SK1-3 can be selectively blocked by apamin and Scyllatoxin [28], [29], [30], though with varying potency. Furthermore, SK-channels are essential for pacemaker-potentials in the neuronal and cardiac system, neuronal excitability, neurotransmitter release and synaptic after-hyperpolarization (AHP), and play important roles in multiple cellular functions, e.g., cell cycle regulation [31], [32], [33], or mesenchymal stem cell proliferation [34]. SK3 molecules in particular are known to mediate the AHP and regulate transmitter release in the presynaptic compartment. The distribution of SK3 in the adult brain is focused to sites of a relatively high density of neurons, such as the substantia nigra and the nucleus subthalamicus [25], [35], implicating an influence on neuronal function in Parkinson's disease [36].

In this study we provide evidence that the SK3 channel is a direct interaction partner of the Abelson-interacting protein 1 (Abi-1) and the neuronal Wiskott-Aldrich-syndrome related protein (nWASP), known to play a pivotal role in cytoskeletal rearrangement [18]. The interplay of this trimeric complex seems to specifically regulate the local rearrangement of the actin cytoskeleton and thereby inducing the formation of small filopodia. The identification of the physiological interplay of these components is not only important for neuronal reshaping of synapses and the protrusion of neurites, but also seems to play a role in the activation, migration and differentiation of neural stem cells during embryogenesis and adult neurogenesis.

## Materials and Methods

### Ethics Statement

All animal experiments were performed in compliance with the guidelines for the welfare of experimental animals issued by the Federal Government of Germany, the National Institutes of Health and the Max Planck Society (Nr. O.103).

### Cell Culture

Neural stem cells were prepared as follows. Adult pregnant Sprague-Dawley rats were anaesthetized and sacrificed with 100% CO_2_, the embryos (embryonic stage E14.5) were removed from the uterus and placed into ice-cold Hank's balanced salt solution (HBSS) supplemented with 1% penicillin/streptomycin and 1% glucose (all from Invitrogen, Karlsruhe, Germany). Their brains were removed and the midbrain was then aseptically prepared. The meninges were carefully removed from the tissue after preparation of the midbrain area. For expansion of neurospheres, tissue samples were incubated in TrypLE Express (Invitrogen) for 10 min. at RT, incubated in DNAse I (40 mg/ml; Sigma-Aldrich, Steinheim, Germany) for 10 min at 37°C, and homogenized to a quasi single cell suspension by gentle triturating with a fire polished pasteur pipette. The cells were added to 25-cm^2^ flasks (2×10^6^ viable cells per flask) in serum-free medium containing 63% DMEM high glucose, 32% Ham's F12, 1% glutamate, 2% B27 supplement, 1% penicillin/streptomycin and 1% non essential amino acids (all from Invitrogen), supplemented with 20 ng/ml of the mitogen EGF (Sigma-Aldrich). Cultures were placed in a humidified incubator at 37°C and 5% CO_2_, 95% air (21% O_2_). After 4 to 7 days, sphere formation was observed. The medium was changed once a week while growth factor was added twice a week. The neurospheres were expanded for additional 3 weeks (in total 3 to 5 passages) in suspension before adherent growth was initiated by plating the cells (about 20.000 cells/well) with the same media without EGF, supplemented with 5% serum replacement (Invitrogen) onto poly-L-lysine (PLL)-coated glass cover slips in 24-well plates. All following studies were then performed 1–24 hours after transferring of the cells to the well-plates.

Cell culture experiments of rat hippocampal primary neurons (embryonic day-18; E18) were performed as described previously [37]. In brief: After preparation the hippocampal neurons were seeded on poly-l-lysine (0.1 mg/ml; Sigma-Aldrich) coated coverslips at a density of r 2×104 cells/well. Cells were grown in Neurobasal medium (Invitrogen,), complemented with B27 supplement (Invitrogen), 0.5 mM L-glutamine (Invitrogen, Karlsruhe, Germany), and 100 U/ml penicillin/streptomycin (Invitrogen) and maintained at 37°C in 5% CO2.

Cos7-cells, (obtained from DSMZ, Braunschweig, Germany) were maintained in Dulbecco's modified Eagle's medium (DMEM) with high glucose (Invitrogen), supplemented with 10% (v/v) fetal calf serum and 2 mM l-glutamine without antibiotics. Cells were grown on commercially available chamber-slides (Nunc, Wiesbaden, Germany) treated with poly-l-lysine (0,1 mg/ml; Sigma-Aldrich).

### Transfection experiments

Hippocampal cells and NSCs were transfected on the days indicated, using Lipofectamine 2000, according to the manufacturer's recommendation (Invitrogen). COS7 cells were transfected using the transfection-agent Fugene (Roche, Mannheim, Germany) according to the manufacturer's recommendations. At 22 hour post-transfection, cells were fixed with 4% paraformaldehyde and processed for indirect immunofluorescence.

### *In situ* hybridization

*In situ* hybridization was performed essentially as described previously [38]. Transcripts encoding SK3 were detected with a S35 labeled cDNA antisense oligonucleotide (ccaagcaggatgatggtggataaactgataaggc)) purchased from MWG-Biotech (Ebersberg, Germany) directed against the 3′ end of the mRNA.

### RNA interference (RNAi) experiments

Knockdown of SK3 was achieved by RNAi following published methods using the pSUPER vector (OligoEngine, Seattle, WA). For this plasmid-based RNA inhibition of SK3 the following complementary oligonucleotides were annealed and inserted into the Hind III/Bgl II sites of the pSUPER vector: 5_GAT CCC CAG GCT ACA GAC AAG AGG AAT TCA AGA GAT TCC TCT TGT CTG TAG CCT TTT TTG GAA A and 3_ AGC TTT TCC AAA AAA GGC TAC AGA CAA GAG GAA TCT CTT GAA TTC CTC TTG TCT GTA GCC TGG G. NSCs were transfected with this construct (RNAi-SK3) Control cells were obtained by transfecting the empty pSuper vector or a scrambled RNAi construct directed against luciferase [39].

### Overexpression constructs

All GFP/RFP/myc-constructs were generated with the described proteins fused N-terminally to a GFP (green fluorescent protein), RFP (red fluorescent protein) or myc-tag (N-EQKLISEEDL-C). SK3-iRES was generated with the full length rat SK3 cDNA into the vector Living Colors® pIRES2-AcGFP1-Nuc (Clontech, Mountain View, USA), which harbors the gene of interest separated from a nuclear GFP by an IRES-site.

### Semi-quantitative real-time one-step RT-PCR

Semi-quantitative real-time one-step RT-PCR was carried out using the LightCycler System (Roche) and amplification was monitored and analyzed by measuring the binding of the fluorescence dye SYBR Green I to double-stranded DNA. 1 µl of total RNA was reversely transcribed and subsequently amplified using QuantiTect SYBR Green RT-PCR Master mix (Qiagen, Hilden, Germany) and 0.5 µM of both sense and antisense primers. Tenfold dilutions of total RNA were used as external standards. Internal standards and samples were simultaneously amplified. After amplification, melting curves of the RT-PCR products were acquired to demonstrate product specificity. Results are expressed relative to the housekeeping gene hydroxymethylbilane synthase (HMBS). Primers for Abi-1, Cofilin, nWASP, SK3 and HMBS were purchased as validated primer pairs (Quantitect primer assay, Qiagen).

### Western blotting

Protein sample concentrations were determined by the amido black protein assay. Equal concentrations of 10 µg protein were loaded per lane and separated by standard SDS polyacrylamid electrophoresis using a polyacrylamid gel with 10% total monomer concentration. Electro-blotting onto nitrocellulose membranes was conducted by standard protocols. Rabbit anti-SK3 specific antibodies (Alomone labs, Jerusalem Israel) were diluted 1∶400 of the recommended stock solution. Mouse anti-GFP antibodies diluted 1∶3000 (Clontech), mouse anti-Abi-1 diluted 1∶500 (MBL, Woburn, USA), mouse anti-Nestin diluted 1∶500 (BD Biosciences, New Jersey, USA), mouse anti-β-Actin diluted 1∶10000 (Sigma-Aldrich), rabbit anti-nWASP diluted 1∶100 (Santa Cruz, Santa Cruz, USA). The ECL detection kit (Amersham Pharmacia, Munich, Germany) was used for immune detection according to the manufactures protocol.

### Pull-down experiments

SK3, Abi-1 constructs (Abi-1 SH3 represents the SH3 domain of Abi-1 alone; Abi-1-d-SH3 is an Abi-1 construct lacking the SH3 domain) and nWASP were transfected in COS7 cells and protein was immobilized on µMACS Micro beads (Miltenyi, Bergisch Gladbach, Germany) coupled with the specific antibodies. Subsequent experiments were performed according to the manufacturer's protocol.

### Immunocytochemical detection

Immunocytochemistry was performed as described in [40]. In brief, cultured cells were fixed with 4% paraformaldehyde (PFA)/1.5% sucrose/PBS for 15 min at room temperature (RT). After washing 3 times with 1x PBS for 5 min at RT the cells were permeabilized for 3 minutes on ice in a buffer containing 0.2% Triton-X-100/0.1% Na-Citrate/PBS and washed again 3 times with 1xPBS. Blocking was performed with 10% FCS/PBS for 1 h at RT followed by incubation with the primary antibodies, diluted in PBS, for 1 hour. After 3 further washing-steps in PBS the cells were incubated with the secondary antibody conjugates for 45 min at RT, washed 3 times with 1xPBS and then with aqua bidest for 3 min and mounted in vectashield aqueous mount (Vector, USA). Cell nuclei were counter stained with 4,6-diamidino-2-phenylindole (DAPI). FM4-64 (Invitrogen) staining was performed according to the manufacturer's protocol. In brief, cover slips were stained in the FM4-64 FX solution for 1 min on ice before fixation and conventional immunocytochemical processing. The following primary antibodies were used: rabbit anti-SK3 diluted 1∶200, rabbit (Alomone Labs), mouse anti-Nestin monoclonal diluted 1∶500 (BD Biosciences), mouse anti-Abi-1 diluted 1∶250 (MBL), mouse anti-myc antibody diluted 1∶500 (Invitrogen), rabbit anti-PSD95 diluted 1∶1000, rabbit anti-SV2 diluted 1∶300 (both abcam, Cambridge, USA) and rabbit anti-nWASP diluted 1∶500 (Santa Cruz) or chicken anti-nWASP diluted 1∶500 (abcam); fluorescence labeled secondary antibodies were Alexa Fluor® 488 (green, used filter set: excitation BP 450 – 490, FT 510, emission BP 515 - 565), Alexa Fluor® 568 (red, used filter set: excitation BP 534 nm–558 nm, FT 560, emission BP 575 - 640) and Alexa Fluor® 647 (magenta used filter set: excitation BP 610 nm–670 nm, FT 660, emission BP 640–740 (all from Invitrogen) all diluted 1∶500. Images were captured using an upright fluorescence microscope (Imager Z1, Zeiss, Oberkochen, Germany) equipped with an Apotome and a Zeiss CCD camera (16 bits; 1280×1024 pixels per Image), and analyzed using Axiovision software (Zeiss). Nuclei were stained with DAPI (blue).

### Statistical analyses and quantification

If not stated otherwise, standard errors of the mean (SEM) are indicated by error bars. Levels of significance were calculated with the unpaired student *t*-test or with the ANOVA model for multiple-group comparisons with post-hoc *t*-test and Bonferroni adjustment. *P*-values of <0.05 were considered significant. Calculations of significance were done with GraphPad Prism 5 (GraphPad Software, Inc., La Jolla, CA, http://www.graphpad.com) or StatView 5.0 (SAS, Chicago, USA). All experiments were performed n = 3 independent experiments and preparations. Quantification of cellular projections was performed according to [19]. In morphological studies of neural stem cells filopodia were considered when processes were unbranched, stained positive by the actin dye phalloidin and extended directly from the cell body. At least 20 cells were chosen randomly for quantification from at least three independent experiments for each condition. The circumference was calculated and all visible filopodia were counted per cell. Morphometric measurements were performed using the upright Axiovision Zeiss microscope with a 40x magnification and the interactive measurement module of the Axiovision software. The total number of filopodia was expressed as density per 1 µm length of cell circumference.

### Application of compounds

The following substances were used: 1-EBIO (1-ethyl-2-benzimidazolinone) (TOCRIS, Bristol, UK) was diluted to a final concentration of 1 mM in the media throughout the experiments. Apamin was diluted to a final concentration of 100 nM (Sigma-Aldrich). Wiskostatin was diluted to a final concentration of 5 µM (Calbiochem, San Diego, CA).

### Subfractionation protocol

Subfractionation of rat whole brain was performed according to [41] with minor modifications. In brief, tissue from 21 day old Sprague-Dawley rats was homogenized in homogenization buffer (320 mM sucrose, 5 mM HEPES, pH 7.4) containing protease inhibitor mixture (Roche). Cell debris and nuclei were removed by centrifugation at 1000×g. The supernatant was spun for 20 min at 12.000×g resulting in supernatant S2 (soluble fraction) and pellet P2 (membrane-associated fraction). P2 was further fractionated by centrifugation in a sucrose step gradient (0.85, 1.0 and 1.2 M) for 2 h at 200.000×g. For isolation of synaptic junctional proteins, the synaptosomal fraction of the first gradient (Sy) was diluted with 5 volumes of 1 mM Tris pH 8.1 and stirred on ice for 30 min. After centrifugation for 30 min at 33.000×g, the pellet P3 was resuspended in 5 mM Tris pH 8.1 and once again fractionated by centrifugation in a sucrose gradient for 2 h at 200.000×g. The 1.0/1.2 M interphase (synaptic junctions, SJ) was suspended in 320 mM sucrose, 0.5% Triton X-100, 5 mM Tris pH 8.1, stirred on ice for 15 min and centrifuged for 30 min at 33.000×g resulting in the first PSD pellet (PSD I). For additional purification, the PSD I pellet was resuspended in the same buffer as the synaptic junctions, stirred on ice for another 15 min and centrifuged for 30 min at 33.000 g finally resulting in the PSD II pellet (PSD II).

## Results

### Neuronal expression of SK3 channels in early brain development

**Figure 1:**
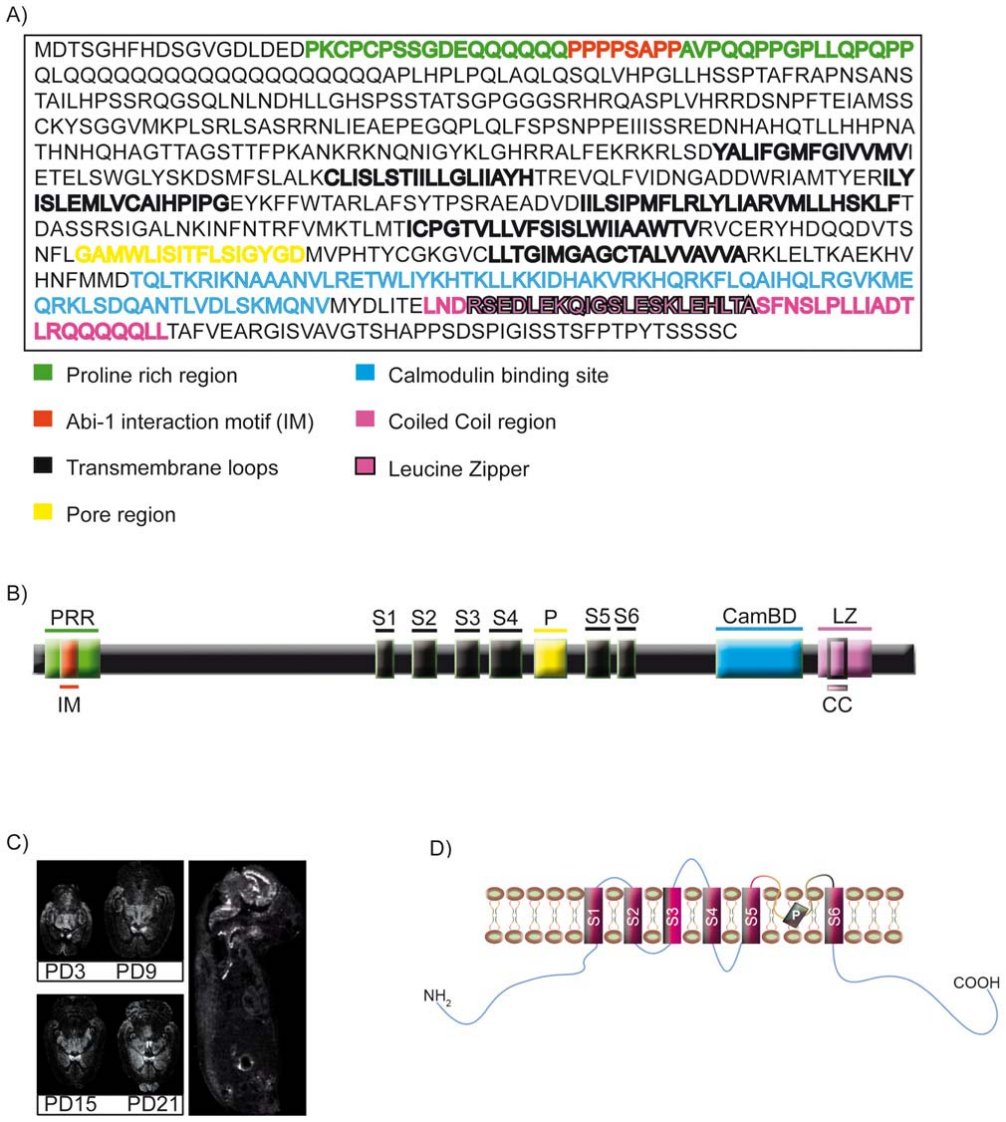
Structure and expression of the SK3 channel. (A,B) Domain structure and amino acid sequence of the rat SK3 channel. An N-terminal proline rich region (green) is indicated that harbors the interaction domain with the SH3 domain of Abi-1 (red). Bold letters indicate the transmembrane domains that are organized around the pore region (yellow). At the C-terminus the calmodulin binding site (light blue) as well as a coiled coil domain (purple) with a leucine zipper motif (purple in black frame) is located. (C) In situ hybridization experiments of a whole body embryo section at developmental stage day 20 and during rat brain development show that the SK3 channel transcripts are nearly exclusively expressed in brain. In embryonic tissue the mRNA is especially found in the subventricular zone, during brain development the transcripts are densely expressed in the dentate gyrus, the olfactory bulb, caudate putamen and in thalamic nuclei. (D) This schematic drawing illustrates the intramembranous localization of the protein showing that both the C- and the N-terminus are intracellular.

**Figure 2:**
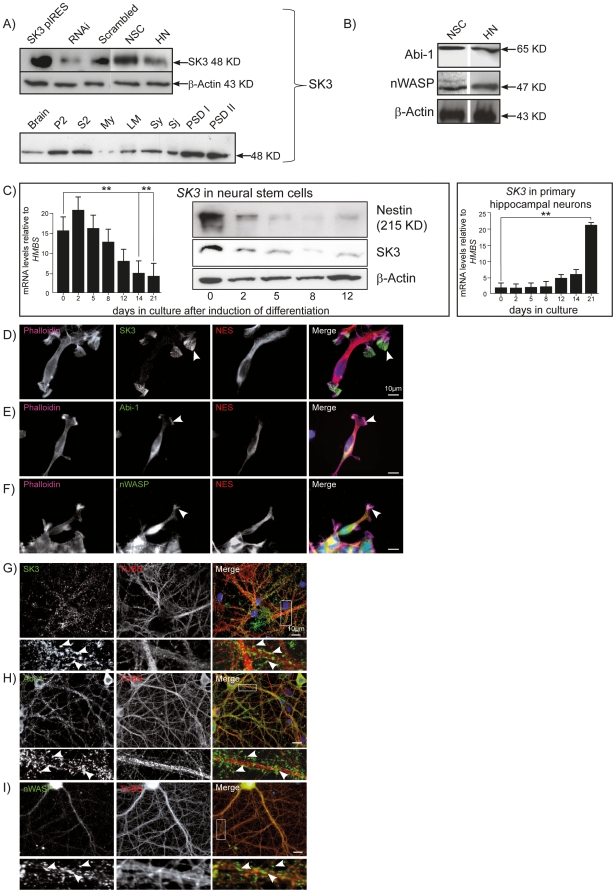
Expression of SK3 channels, Abi-1 and nWASP in neuronal stem cells (NSCs) and in primary hippocampal neurons (HNs). (A) Western Blot analysis of SK3 channel transfected NSCs (SK3 pIRES) indicates that the SK3 channel antibody readily recognizes the overexpressed protein. NSCs transfected with the RNAi construct show a decrease of SK3 protein compared to NSCs transfected with a scrambled RNAi construct and untransfected cells. The SK3 channel protein can also be detected in hippocampal neurons. Subfractionation of brain tissue reveals that the protein is enriched towards the postsynaptic density fraction (PSD). (B) nWASP and Abi-1 are detectable in NSCs as well as in hippocampal neurons. (C) Light cycler analysis of SK3 channel mRNA in NSCs and hippocampal neurons reveals that SK3 transcript levels are downregulated after induction of differentiation in NSCs but show a steep increase in later steps of hippocampal neuron maturation. *P*-values from ANOVA for multiple-group comparison are 0.0011 for NSCs and <0.0001 for hippocampal neurons (only selected post-hoc *t*-test *P*-values are displayed for clarity, * represents *P*<0.05, ** *P*<0.01). Western Blot analysis show a decrease of Nestin protein after plating of NSCs representing ongoing differentiation accompanied by a decrease of SK3 proteins. (D–F) Immunohistochemical staining of neural stem cells characterized by the stem cell marker NES (red) shows the localization of SK3 channels, Abi-1 and nWASP (green) co-localizing with the actin cytoskeleton as labeled by phalloidin (magenta, arrowheads). Nuclei are visualized by DAPI staining (blue). (G–I) In hippocampal neurons all three antigens (green) are mainly localized within the dendritic compartment and are highly enriched within hippocampal spines and postsynaptic densities (PSDs). Tubulin beta 3 (Tubb) is stained in red, DAPI (blue) is used to label nuclei. P2 membrane associated fraction; S2 soluble fraction; My myelin fraction; LM light membranes; Sy synaptosomal fraction; Sj synaptic junctions; PSDI, PSDII postsynaptic density fractions. Scale bars as indicated.

Functional SK channels are tetrameric and can be composed of 3 different α-subunits in a homomeric or heteromeric fashion and can also include an isoform of SK2 with an extended amino terminus [42]. SK3 channel proteins exhibit several domains, including a proline rich region (PRR), six transmembranous loops (S1-S6), a pore region (PR), a calmodulin binding region (CamBd) and a leucine zipper within a coiled coil domain (CC, LZ) (Fig. 1A;B;D). The PRR, CamBd and the LZ are localized intracellularly. In the rat embryo, the SK3 channel mRNA is strongly expressed, predominantly in brain, already early in development (embryonic day 19) and shows a neuronal expression pattern within the cerebellum, caudate putamen, dentate gyrus of the hippocampus, thalamic nuclei and in the olfactory bulb in adult animals. Western blot analysis of NSCs overexpressing SK3, NSCs depleted of SK3 by RNAi, NSCs expressing a scrambled RNAi construct, untransfected NSCs or hippocampal neurons show SK3 protein bands in different strength. NSCs and hippocampal neurons both express the actin modulating proteins Abi-1 and nWASP. The detection of SK3 channel immunoreactivity in subfractions of rat brain shows that this membrane protein is strongly enriched towards the postsynaptic density fraction (PSD) (Fig. 2A,B). mRNA concentrations of the SK3 channels are dynamic in NSCs and hippocampal neurons during development. Both protein and mRNA levels show a decrease of SK3 in NSCs after initiation of differentiation, shown by a protein and mRNA decrease of the neural stem cell marker Nestin and increase of the neural markers *TUBB3* for neurons and *GFAP* for glial cells (Fig. S1E). mRNA levels increase during the maturation of hippocampal neurons especially between d14 and 21 in culture (Fig. 2C). This might represent the known functional role of SK3 during late phase of neuronal differentiation and in mature neurons. The abundance and function of SK3 in working neuronal circuits has already been shown by several groups. Most probably, the increase in transcript levels of SK3 points to an increased function in synaptic hyperpolarization. At later time points SK3 is therefore specifically found in the presynaptic specialization. Immunocytochemical staining of stem cells show the localization of all three proteins at similar compartments such as lamellipodia and membrane bound structures. While SK3 channels are predominantly targeted to the leading edge of lamellipodia and filopodial, Abi-1 and nWASP show an additional distribution in the cytoplasm (Fig. 2D–F). In hippocampal neurons the proteins are especially enriched within the dendritic compartment where they show the tendency to form immunopositive clusters at spines and postsynaptic densities (Fig. 2G–I). nWASP is more widely scattered in small clusters within the neurons. In young neurons it is not surprising that we could find SK3/nWASP positive clusters only partly co-localizing with markers of internalized vesicles by endocytosis at excitatory synapses. In addition, these immature neurons showed only few mature synapses with rare postsynaptic density protein PSD95 positive PSDs which did co-localize with few clusters that were positive for nWASP and SK3. Synaptic vesicles, which are marking presynapses or preassembled presynaptic proteins, were stained opposed to nWASP/SK3 clusters (Fig. S1A).

### SK3 channels are in a complex with Abi-1 and nWASP

**Figure 3:**
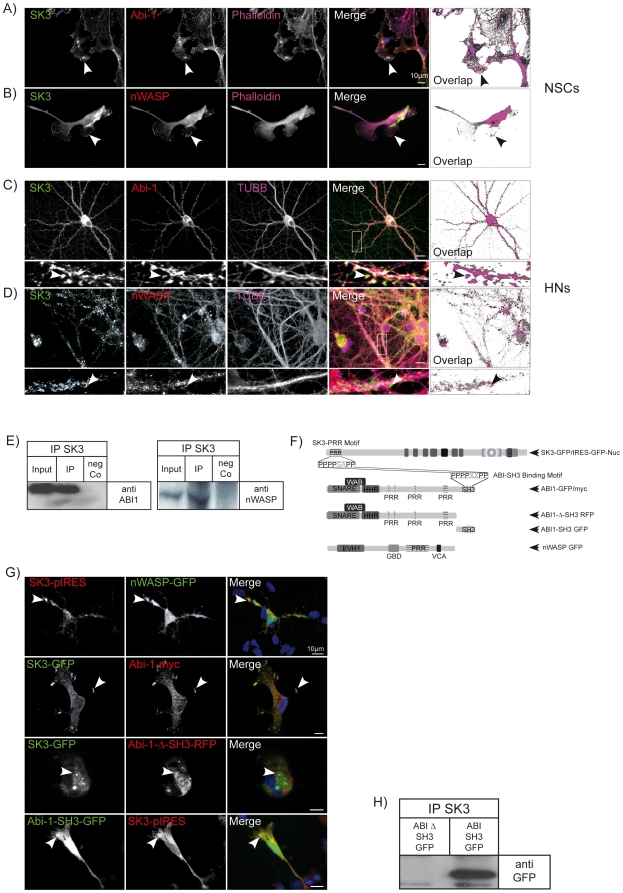
SK3 channels, Abi-1 and nWASP are found in neuronal complexes and colocalize in defined subcompartments of NSCs and hippocampal neurons (HNs). (A-B)Triple staining experiments show that Abi-1 as well as nWASP (red) colocalize with the SK3 channel (green) in distinct lamellipodia like structures of NSCs that are also rich of actin fibers stained by phalloidin (magenta), DAPI (blue) is used to label nuclei. (C–D) In hippocampal neurons SK3 channels are densely targeted to dendritic spines/PSDs and co-localize with nWASP as well as with Abi-1 (see arrows in insets). To visualize the cytoskeleton tubulin antibodies (Tubb, magenta) are used, DAPI staining (blue) show neuronal nuclei. (E) Co-immunoprecipitation experiments show the presence of all three molecules in one neuronal complex also *in vivo*. Magnetic beads loaded with SK3 channels antibodies were employed to precipitate the SK3 channel complex from rat brain. Within this precipitate Abi-1 as well as nWASP can be detected. (F) Schematic drawing of the fusions proteins and Abi-1 deletion constructs used for transfection experiments with their respective domain regions. The SK3 channel codes for a conserved N-terminal proline rich stretch that mediates the interaction with the Abi-1 SH3 domain. (G) Co-transfection of the nWASP with the SK3 channel results in a complete colocalization in NSCs. This is also observed when full length Abi-1-myc is co-transfected with the SK3 channel GFP fusion protein. The co-expression of SK3 channels with an Abi-1 SH3 domain deletion construct (Abi-1 ΔSH3-RFP) results in no co-localization of the proteins while the SK3 channel perfectly co-localizes with the Abi-1-SH3 domain fusion protein. Moreover, the full length SK3 channel protein can co-precipitate the GFP-SH3 domain from the co-transfected cell lysate but fails to bind to the Abi-1 protein that is not expressing the SH3 domain. Scale bars as indicated.

Double immunocytochemical stainings of NSCs and hippocampal neurons show the colocalization of SK3 channels and Abi-1, nWASP respectively, in defined subcompartments. In NSCs the molecules are found in concert with the actin cytoskeleton underneath the membrane of cellular protrusions. In hippocampal neurons the proteins show overlapping localization at spiny protrusions within the dendritic tree (Fig. 3A–D). These spines represent amongst others precursors of synapses. These structures are highly dynamic and are sites of fast changes of the actin cytoskeleton. Immunoprecipitation experiments underline this observation by showing that Abi-1 as well as nWASP are indeed localized in one neuronal complex so that they both can be precipitated by specific SK3 channel antibodies (Fig. 3E). After cotransfection of NSCs with either Abi-1 and/or nWASP and SK3 channel fusion protein both molecules are recruited to identical cellular clusters (Fig. 3G). The cotransfection of Abi-1 deletion constructs strongly supports the hypothesis that the N-terminal proline rich region within the SK3 channel protein mediates the interaction with the Abi-1 SH3 domain (Fig. 3G). The SH3 domain alone shows a perfect co-localization with SK3 channels, the Abi-1 construct without the SH3 domain is diffusely distributed in the cytoplasm and does not co-cluster with SK3 channel proteins. This is also shown by co-immunoprecipitation experiments from transfected COS cells where the SK3 channel protein is bound to the precipitated Abi-1 SH3 domain alone (Fig. 3H).

### SK3 channels in concert with nWASP are effectively inducing filopodia formation in NSCs

**Figure 4:**
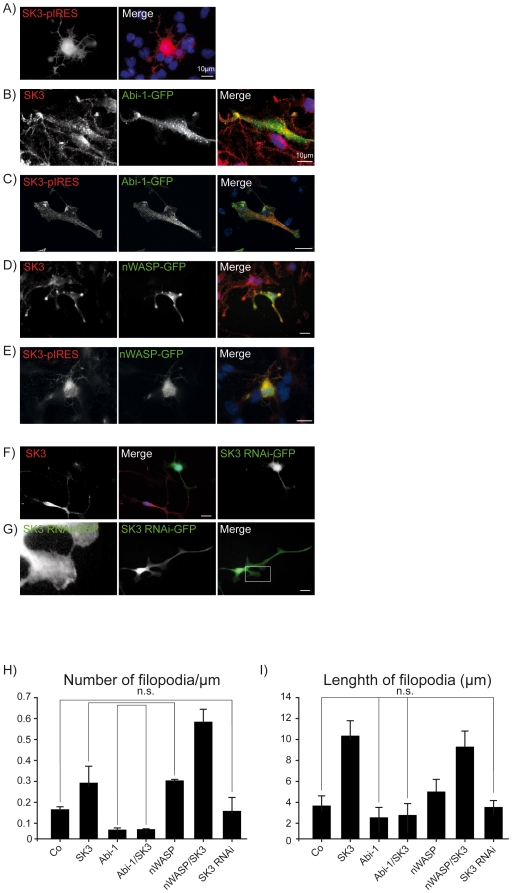
SK3 channel, nWASP and Abi-1 protein levels alter NSC morphology. (A–G) Overexpression of SK3 channels in NSCs significantly triggers long filopodia formation in NSCs while Abi-1 overexpression downregulates filopodia number and length (B). (C) Co-expression of SK3 channels and Abi-1 results in an NSC morphology that is identical to Abi-1 overexpression alone. (D) nWASP transfection strongly induces filopodia formation and this effect can be even enhanced by double transfection of nWASP together with SK3 channels (E). Downregulation of SK3 channels in NSCs by an RNAi construct that efficiently diminishes SK3 channel protein concentrations (F) does not significantly influence filopodia number and/or length of filopodia (G). Scale bars as indicated. (H–I) Statistical analysis of NSC morphology (number of filopodia per 1 µm/length of filopodia) after the above described transfection experiments. *P*-values from ANOVA for multiple-group comparison are <0.0001 for number of filopodia (H) and <0.0001 for length of filopodia (I). All groups were significantly different except the groups marked with bars. Non-significant Post-hoc *t*-test *P*-values are displayed in the diagrams (n.s.).

Overexpression of SK channels in NSCs changes the morphology of neural stem cells and induces the rapid formation of filopodial processes. Interestingly the overexpression of Abi-1-GFP had an opposite effect and drastically reduced the formation of filopodia in stem cells (Fig. 4A–C,H,I). Overexpression of nWASP, however, resulted in significantly increased levels of filopodia as observed after SK3 channel overexpression. This effect was even enhanced in an additive manner by double transfection with nWASP and SK3 channels. The property of SK3 channels to induce long filopodial processes was completely abolished when SK3 channels were cotransfected together with the GFP-Abi-1 construct. Here, the effect of Abi-1 was shown to be dominant since filopodia were reduced to numbers that were seen in NSCs transfected with Abi-1 GFP alone (Fig. 3C–E,H,I). The transfection of an SK3 channel RNAi construct into NSCs that effectively diminished the SK3 protein content did not significantly change the number and length of filopodia in NSCs (Fig. 4F–I). As the endogenous expression of SK3 in NSCs is strong, we measured overexpression levels by grayscale measurements in stainings and found levels elevated 24 h after transfection approximately 5–6 times (Fig. S1).

### Activation or inhibition of SK3 channels effectively alter NSC morphology

**Figure 5:**
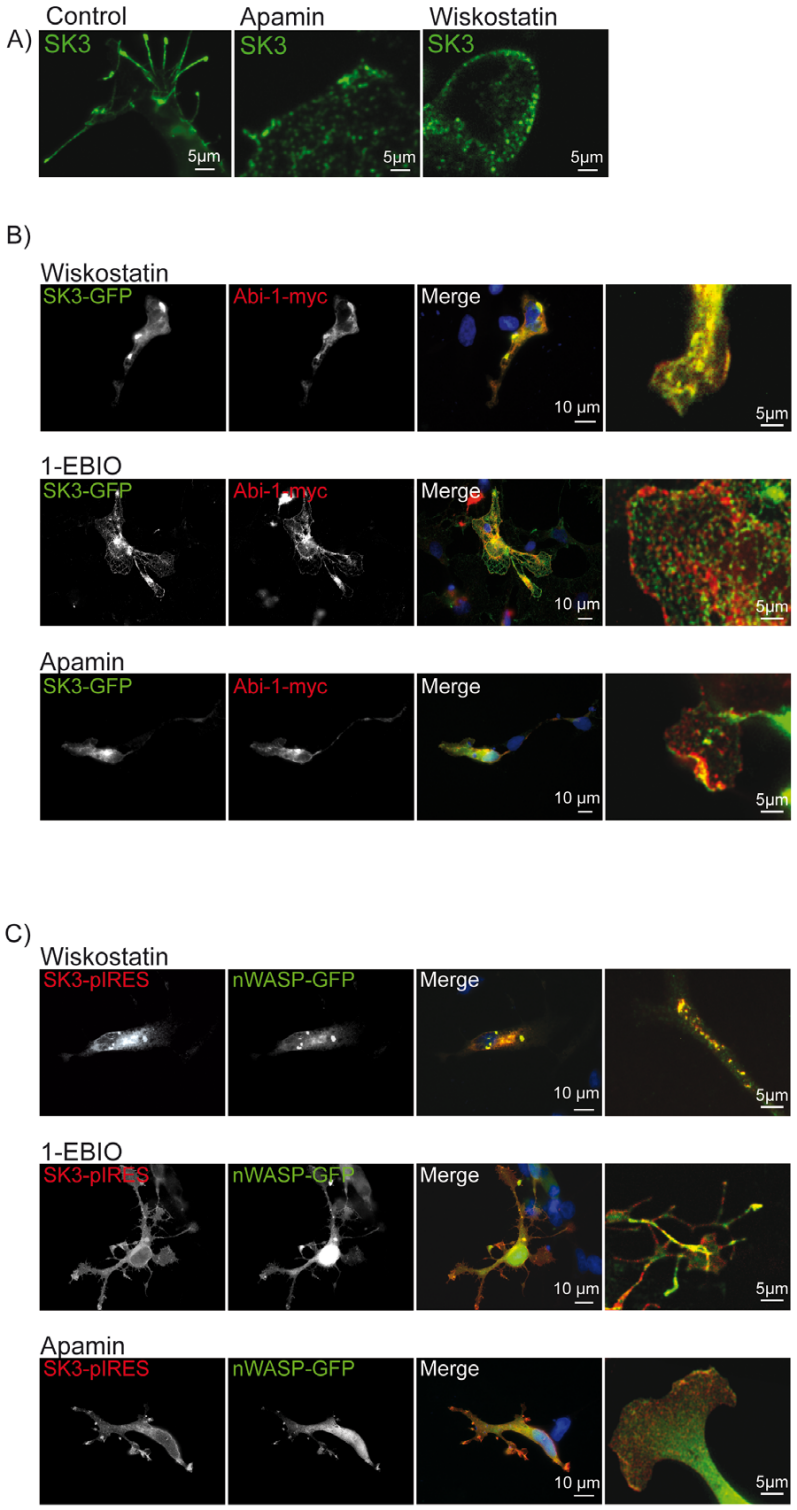
Pharmacological treatment of NSCs with 1-EBIO, wiskostatin and apamin. Application of the SK3 channel opening substance 1-EBIO shows the rapid outgrowth and enlargement of filopodia with a translocation of the channel into the newly build processes in SK3 channel overexpressing NSCs. Apamin treatment that leads to a blockage of SK3 channels as well as the application of wiskostatin drastically reduces filopodia formation. (B) Double transfection of NSCs with SK3 channels and Abi-1 results in the co-localization of both proteins in the cell cytoplasm. The application of wiskostatin and apamin to the cells induced the translocation of the proteins to small microcompartments. In contrast, the SK3 channel activator 1-EBIO induced the rapid outgrowth of numerous large lamellipodia that show a netlike arrangement of SK3 channel immunoreactivity. (C) The double transfection of SK3 channels and nWASP resulted in a morphology of NSCs that was characterized by larger filopodial extensions that were lost after application of wiskostatin, enhanced and more elaborated under the influence of 1-EBIO and altered towards lamellipodia after apamin application. Scale bars as indicated.

In further investigations we overexpressed SK3, Abi-1 and nWASP in combinations as indicated and directly interfered with SK3 channel physiology. We applied 1-EBIO that enhances SK3 channel activity, as well as apamin, a toxin that blocks these channels. Moreover, we applied wiskostatin, a cell permeable carbazole derivate that inhibits nWASP mediated actin polymerization. 1-EBIO exerted a dramatic effect on SK3 channel transfected cells developing long filopodia filled with SK3 channel protein. Wiskostatin inhibited the development of cellular protrusions and rapidly lead to a disorganization of the cytoskeleton (Fig. 5A). In NSCs that were double transfected for SK3 channels and Abi-1 (characterized by very few filopodia, see Fig. 4), the application of 1-EBIO resulted in the formation of large netlike lamellipodia, most likely due to the branching activity of Abi-1. Apamin had no additional effect on NSC structure but did also not influence the co-localization of the transfected fusion proteins. Wiskostatin solely lead to the already described disassembly of the cytoskeleton (Fig. 5B). Finally, the effects after co-transfection of SK3 channels and nWASP (very potent in inducing protrusions; see Fig. 4) could further be enhanced by 1-EBIO application resulting in numerous and long filopodial together with large laminar cell compartments. Wiskostatin abolished the formation of protrusions at all; apamin diminished the number of NSC filopodial (Fig. 5C).

### The number of neurites in developing hippocampal neurons is depending upon protein levels of SK3-channel, Abi-1 and nWASP

**Figure 6:**
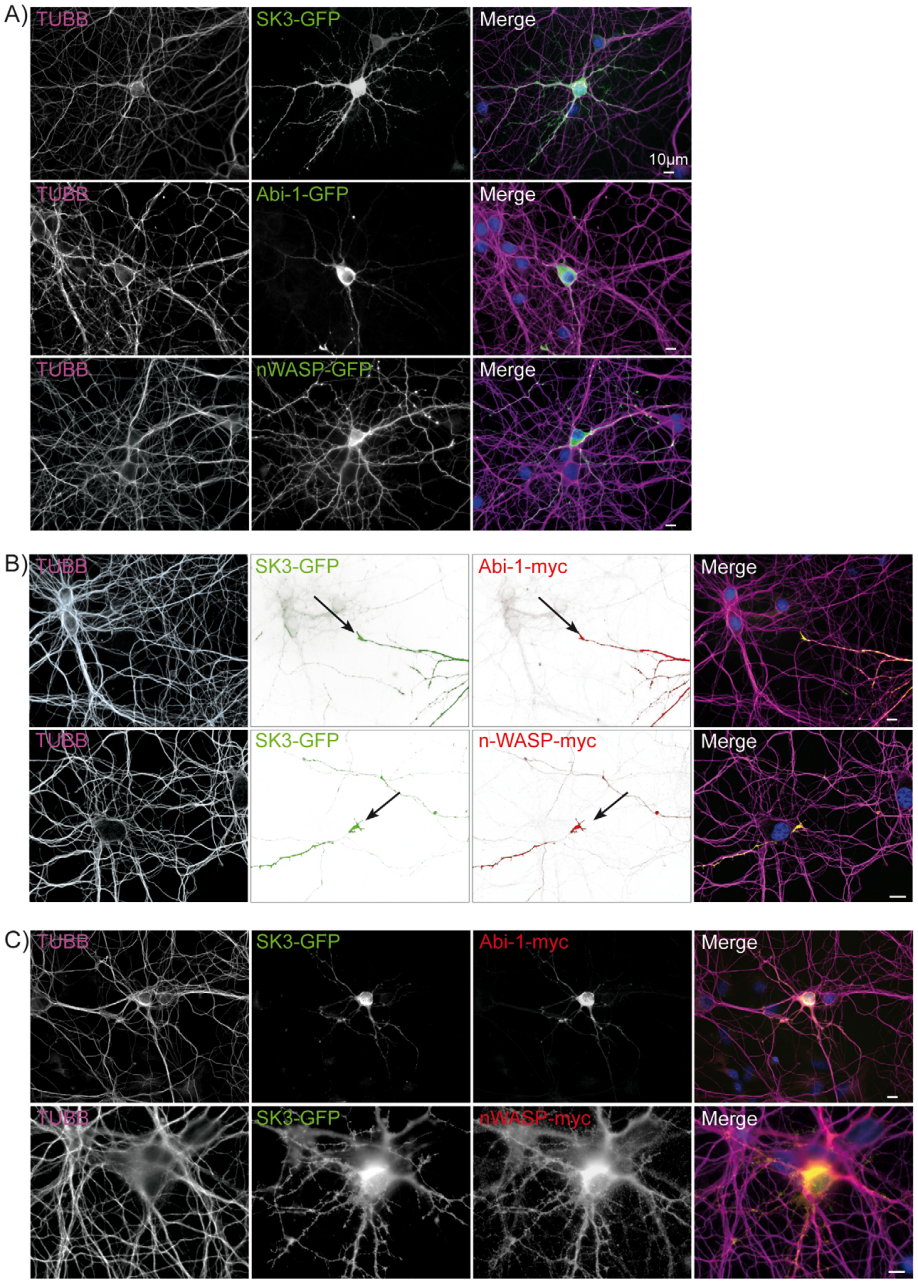
SK3 channel, nWASP and Abi-1 protein levels also alter hippocampal neuron morphology. (A) In close homology to the observation in NSCs we found that the overexpression of SK3 channels as well as of nWASP in developing hippocampal neurons is leading to a very complex dendritic tree with numerous secondary and tertiary dendrites. In contrast the Abi-1-GFP fusion protein extremely simplifies the dendritic arbor. (B-C) Overexpression of SK3 channels and Abi-1, nWASP respectively, resulted in a perfect co-localization of both fusion proteins especially in outgrowing neurites. Co-expression of SK3 channels and Abi-1 abolishes the SK3 channel overexpression phenotype while the addition of nWASP to SK3 channel transfected neurons is leading to a multidendritic neuron that is characterized by numerous primary, secondary and tertiary neurites. Nuclei are stained with DAPI (blue). Scale bars as indicated.

Finally, with respect to the results obtained in NSCs, we analyzed the effect of SK3 channels, Abi-1 or nWASP fusion proteins on developing hippocampal neurons. The single transfection of the respected plasmids resulted in a neuronal phenotype that was quite similar to the observations in NSCs. Overexpression of SK3 channels and –even more- nWASP induced a very complex dendritic tree with numerous secondary and tertiary dendrites. Abi-1 expression in neurons had an opposite effect showing a very simplified dendritic arbor (Fig. 6A). Double transfection of SK3 channels with either Abi-1 or nWASP resulted in a perfect co-localization of the proteins especially in outgrowing neuronal protrusions (Fig. 6B). While SK3 channel expression could not rescue the Abi-1 overexpression phenotype, the co-transfection of nWASP and SK3 channels tremendously enhanced the formation of primary, secondary and tertiary dendrites (Fig. 6C).

## Discussion

In this study, we further characterized an effective signaling cascade that is able to transduce Ca^2+^ signals via SK3 channels into structural changes of cellular protrusions in neural stem cells and developing hippocampal neurons. This pathway is based on a trimeric complex composed of SK3 channels and the actin binding proteins Abi-1 and nWASP. Small conductance Ca^2+^ activated K^+^ channels are gated solely by the binding of calmodulin (CaM), which is constitutively linked to the channel [27], mediating afterhyperpolarization that follows single or trains of action potentials [43]. Several studies have shown that SK1, SK2 and SK3 channels can be detected in neurons of all brain regions with overlapping patterns of expression [44], [45], [46]. In the adult brain, SK3 channels are mainly found in subcortical regions. This finding is supported by our developmental in situ hybridization data that show a strong transcriptional activity especially in thalamic nuclei, the hippocampus and in the caudate putamen. In contrast, in fetal brain stages the mRNA is highly expressed in the subventricular region of the developing brain supporting an important role of this molecule in maturation processes of neurons from neuronal stem cells. This hypothesis is further supported by the quantification of SK3 channel mRNA during differentiation. Here, mRNA concentrations steadily decrease in the process of neuronal specification indicating that SK3 channel activity might also important for neural stem cell identity. Immunohistochemical analysis of the subcellular distribution of the SK channels in neurons revealed clear differences especially with respect to pre- and/or postsynaptic localization of the proteins. SK3 channels were shown to be mostly presynaptic in the adult mouse brain as well as in mature hippocampal cultures from mice [45], [47]. These data are in part contradictory to our observations in NSCs and in developing hippocampal neurons. Still, as we found SK3 to be important for cytoskeletal development, a varying function of this channel protein during cell and organ development might explain the differing localization in the cell. In NSCs as well as in young hippocampal neurons we provide several lines of evidence that SK3 channels build submembranous clusters with nWASP and Abi-1 in NSCs and co-localize in spines of developing dendrites in rat hippocampal neurons. Moreover, SK3 immunoreactivity was readily detectable in PSD subfractions and the SK3 protein was found to coprecipitate with endogenous nWASP and Abi-1 from rat brain lysate. All expression constructs transfected into young neurons are targeted to the postsynaptic compartment. It can well be that the observed differences are due to a time dependent shift of the protein from the postsynaptic to the presynaptic compartment. However, especially with respect to the proposed role of SK channels in synaptic plasticity and memory formation [48], [49], [50], [51], these novel data have to be taken into close consideration.

Our investigations show by several lines of evidence that SK3 channels are part of a functional complex that –besides calmodulin- is at least composed of nWASP and Abi-1. This complex acts in defined microcompartments and creates a local signaling cascade associated with SK3 channel activity. We could show by overexpression of the proteins and/or activation vs. inhibition of SK3 channels that the fast alterations of NSC or hippocampal neuron morphology is synergistically induced by SK3 channels and nWASP. The additional activation of SK3 channels by EBIO-1 provoked the formation of extremely large cellular protrusions. Moreover, the inhibition of nWASP by wiskostatin prevented the formation of any filopodia even in transfected NSCs. In hippocampal neurons the double transfection of SK3 channels and nWASP extremely induced especially quartary dendrites or spines. This is in accordance to the observations by Wegner et al., (2008) who found that nWASP regulates spine and synapse formation in cultured neurons [52]. The neuronal distribution of nWASP is associated with the broad function of this protein in several complexes of the cytoskeletal machinery throughout the immature neuronal cell. In mature functional neurons it was described at excitatory synapses co-localizing to sites of endocytosis. Additionally, nWASP was shown to co-localize with PSD95 stainings, opposing stainings of synaptic vesicles of the presynaptic specialization. Still in young neurons where not only spines or synapses are built but the dendritic tree is expanded and branched, proteins of the actin machinery are more widely distributed. Therefore all investigated proteins, Abi-1, nWASP and SK3, are present throughout the neuronal submembranous compartment. The blockage of SK3 channels by apamin altered the morphology of the protrusions towards a more lamellipodia like appearance. Interestingly, Abi-1 is able to strongly counteract the SK3 or nWASP effect. In earlier studies the role of Abi-1 in stabilizing the actin cytoskeleton has already been reported [13], [53], however, it is hitherto not completely resolved how this effect is achieved and how it is controlled. Abi-1 is also part of a trimeric complex where it closely interacts with Eps8 and Sos-1. Here Abi-1 has been found to control WAVE2 signaling via the regulation of Rac activity [54], [55]. Moreover, Abi-1 is important for the phosphorylation of protein complexes through the non-receptor tyrosine kinase c-Abl [56]. C-abl, Eps8 and Sos-1 have also been detected within spines and PSDs of excitatory synapses so that these molecules might also be directly or indirectly linked to SK3 channels [57], [58], [59]. nWASP on the other hand is involved in multiple protein-protein interactions which regulate or modulate various cellular mechanisms. These mechanisms include e.g. endocytosis or chemotaxis [60], [61] and, in response, they activate the Arp2/3 complex [62], . It has been reported that nWASP activity is mediated via the c-terminal VCA region of nWASP by binding and activating the Arp2/3 complex [64]. We suppose that SK3 –via its interaction with Abi-1 and nWASP – is modulating signaling cascades, e.g. calcium concentrations in the microcompartment, which in turn leads to a modulation of the actin cytoskeleton modulating complex, e.g. nWASP/Arp2/3. Further investigations should therefore focus on the influence of SK3 channel activity on calcium level modulation and small GTPases especially of the Rac as well as of the Rho family to explain filopodial growth via actin polymerization.

In summary we show that SK3 channels are functional components of an nWASP/Abi-1 complex in cellular subcompartments that regulates the number and complexity of dendrites and spines in neural stem cells as well as in hippocampal culture. Therefore, at least at early stages of development postsynaptic SK3 channel activity can influence neural morphogenesis via the nWASP mediated restructuring of the actin cytoskeleton.

## Supporting Information

Figure S1 **Localization of SK3/nWASP clusters in young neurons (12 DIV), levels of overexpression in SK3 transfected NSCs and proof of differentiation of plated NSCs on mRNA levels.** (A) nWASP (magenta) and SK3 (red) show an opposed staining pattern with SV2 in early synaptic compartments. (B,C) The complex of SK3 (green) and nWASP (magenta) is co-localizing at early synaptic clusters with the postsynaptic density protein PSD95 and the endocytotically integrated vesicle marker FM4-64. (D) Overexpression levels of the pIRES-SK3 construct elevates the levels of SK3 proteins at single cell level. Scale bars as indicated. (E) NSCs differentiate into Neurons and glial cells after plating. mRNA levels of the stem cell marker Nestin (*NES*) decrease while markers of neurons, tubulin beta 3 (*TUBB3*) and glial cells, the glial fibrillary acidic protein (*GFAP*) are increased during differentiation. Nuclei are stained with DAPI (blue). *P*-values from ANOVA for multiple-group comparison are 0.0309 for *TUBB3*, <0.0001 for *GFAP* and 0.0199 for *NES*. Post-hoc *t*-test *P*-values are displayed in the diagrams with * representing *P*<0.05 and ** indicating *P*<0.01 (only selected post-hoc *t*-test *P*-values are displayed for clarity.
(TIF)
