# Supplementary figures and images for: An SK3 Channel/nWASP/Abi-1 Complex Is Involved in Early Neurogenesis
Source: PLoS One. 2011 Mar 25;6(3):e18148. doi: 10.1371/journal.pone.0018148 (PMC3064656; doi:10.1371/journal.pone.0018148)

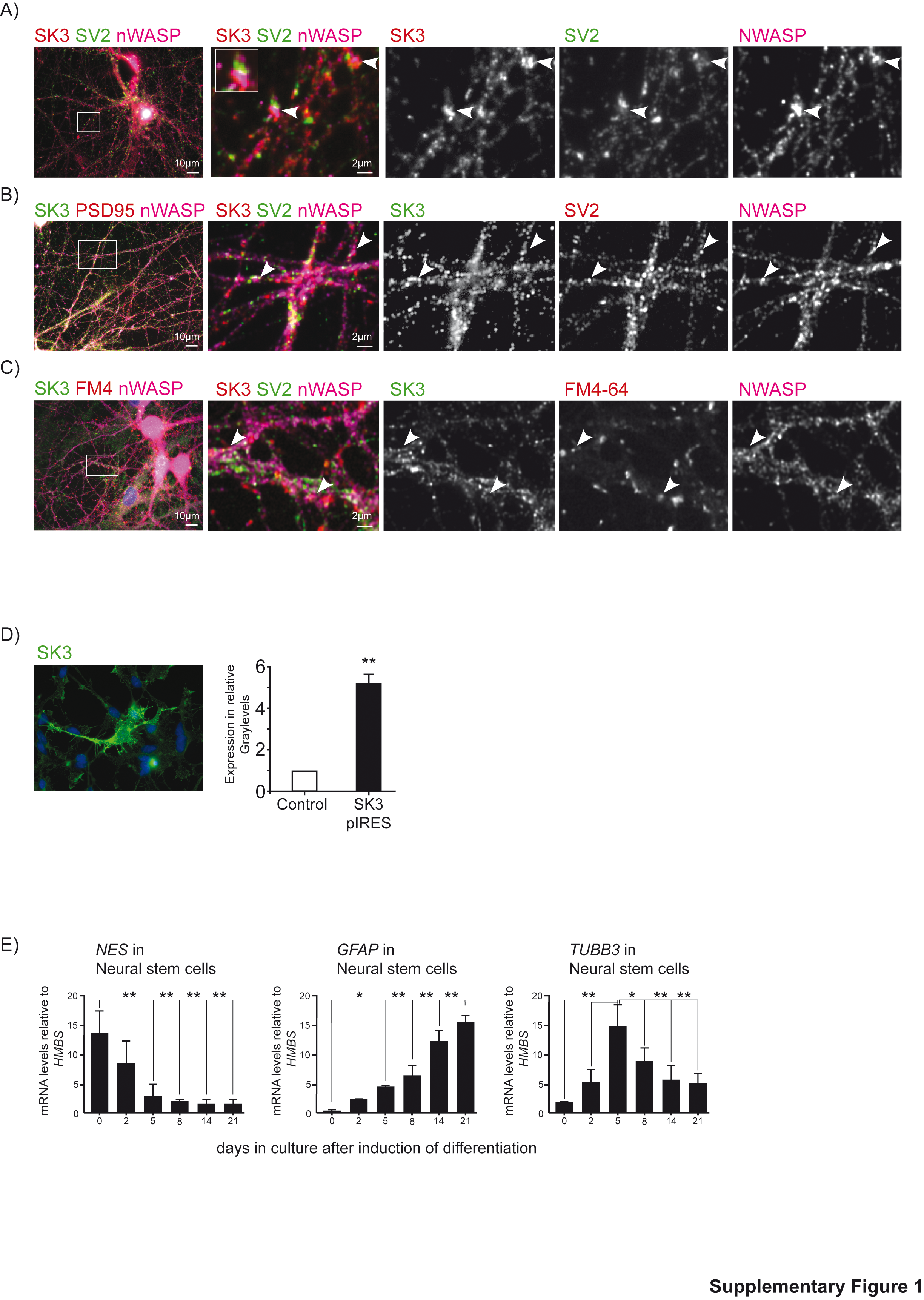

Supplement: Figure S1 — Localization of SK3/nWASP clusters in young neurons (12 DIV), levels of overexpression in SK3 transfected NSCs and proof of differentiation of plated NSCs on mRNA levels. (A) nWASP (magenta) and SK3 (red) show an opposed staining pattern with SV2 in early synaptic compartments. (B,C) The complex of SK3 (green) and nWASP (magenta) is co-localizing at early synaptic clusters with the postsynaptic density protein PSD95 and the endocytotically integrated vesicle marker FM4-64. (D) Overexpression levels of the pIRES-SK3 construct elevates the levels of SK3 proteins at single cell level. Scale bars as indicated. (E) NSCs differentiate into Neurons and glial cells after plating. mRNA levels of the stem cell marker Nestin (NES) decrease while markers of neurons, tubulin beta 3 (TUBB3) and glial cells, the glial fibrillary acidic protein (GFAP) are increased during differentiation. Nuclei are stained with DAPI (blue). P-values from ANOVA for multiple-group comparison are 0.0309 for TUBB3, <0.0001 for GFAP and 0.0199 for NES. Post-hoc t-test P-values are displayed in the diagrams with * representing P<0.05 and ** indicating P<0.01 (only selected post-hoc t-test P-values are displayed for clarity. (TIF) [file pone.0018148.s001.tif]
